# Successful approach for couples having their own genetic offspring using vitrified‐warmed oocytes injected by frozen‐thawed poor quality testicular sperm: A case report

**DOI:** 10.1002/ccr3.5627

**Published:** 2022-03-20

**Authors:** Somayyeh Safari, Hoora Amouzegar, Sareh Ashourzadeh, Elham Hosseini

**Affiliations:** ^1^ Department of Obstetrics and Gynecology, Faculty of Medicine Qom University of Medical Sciences Qom Iran; ^2^ Clinical Research Development Center Forghani Hospital, Qom University of Medical Sciences Qom Iran; ^3^ 48463 Afzalipour Clinical Center for Infertility Kerman University of Medical Sciences Kerman Iran; ^4^ Department of Obstetrics and Gynecology, Mousavi Hospital School of Medicine, Zanjan University of Medical Sciences Zanjan Iran; ^5^ Zanjan Metabolic Diseases Research Center Zanjan University of Medical Sciences Zanjan Iran

**Keywords:** cryopreservation, oocyte, testicular sperm, vitrification

## Abstract

The clinical applications of donated gametes are approved in many countries; however, attitudes toward its application and national legislation in some countries are challenging. The purpose of this study is to report a healthy live birth produced by vitrified‐warmed oocytes and frozen‐thawed testicular sperms to avoid sperm donation.

## Graphical abstract

The application of donated sperm has pros and cons; so, it is highly stigmatized in most nations. Using severe abnormal testicular sperms is a promising approach yet. Infertility treatment should not be bypassed by sperm donation.

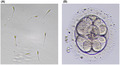

## 1 INTRODUCTION

Todays, by replacing the oocyte slow freezing cryopreservation method with vitrification and adjustment of freezing conditions in the field of assisted reproductive technology (ART), the survival rate of oocytes and embryos has dramatically improved.^1^ Oocyte cryopreservation offers several options, including fertility preservation in women undergoing cancer treatment, for example, chemotherapy or radiotherapy, in egg donation cycles, for women who electively delay childbearing, for a male partner who cannot be present or unable to ejaculate, or in azoospermia case whom testicular sperm extraction (TESE) procedure is failed at the day of ovum pick up.

Successful live births have been observed in vitrified‐warmed oocyte cycles in which oocytes are injected with fresh spermatozoa.^2^ Besides warmed oocyte quality, sperm parameters (morphology and motility) are also a prognostic factor for TESE coupled with intracytoplasmic sperm injection (ICSI) cycle outcomes. Therefore, almost all infertility clinics recommend using donated sperm in such a situation. However, the use of donated sperm has different pros and cons. In addition, it is highly stigmatized in most Islamic nations due to national regulations, religious or other legal, moral, ethical concerns, and social attitudes. For these reasons, some couples are obliged to cross countries or even divorce in order to access donor gametes.^3^

We describe the live delivery of a healthy female infant following the transfer of embryos produced by ICSI using vitrified‐warmed oocytes and frozen‐thawed severely poor quality testicular sperms to avoid sperm donation's consequences.

## 2 CASE PRESENTATION

### 2.1 Patient history

The couple with a history of primary infertility, an unsuccessful ICSI/ET cycle, and severe male factor was referred to University Hospital, Qom, Iran. The woman was 19‐year‐old. Demographic information of the couple, the hormonal profile of the woman on Day 2 of the menstrual cycle, and the history of previous ICSI/ ET cycle are shown in Table 1.

**TABLE 1:** Clinical and demographic data of the couple

Female partner	
Age (year)	19
BMI	20.5
Infertility (year)	3
AMH (ng/µl)	1.3
FSH (mIU/ml)	3.9
LH (mIU/ml)	1.4
Estradiol (pmol/l)	51.1
Previous ICSI cycle	
Number of oocyte	12
MII oocyte	9
Number of embryos	4
Current ICSI cycle	
Number of oocyte	8
MII oocyte	7
Number of embryos	1

Male partner	
Age (year)	28
BMI	26.5
Sperm parameters of fresh sample before cycle	
Count (10^6^)	0.02
Morphology (%)	0
Motility (%)	
Progressive	0
Non‐progressive	10
Immotile	90

The medical record investigation showed that in the previous ICSI/ET cycle in another IVF center, nine MII oocytes were injected with immotile/amorphous spermatozoa of freshly ejaculated semen. Four Day 3 embryos (grade: 6B, 4B, and 3B^2^) were formed; considering the poor quality, all embryos were transferred, with a negative outcome.

After 6 months, semen analysis in our center indicated 0.02 million per ml count with 10% non‐progressive motility and total abnormality in morphology (oligo‐astheno‐teratozoospermia).

### 2.2 Patient management

The case was carefully evaluated. The medical records showed that there was no improvement in semen parameters despite the male partner had received medical treatment such as human menopausal gonadotropin (HMG) plus human chorionic gonadotropin (HCG), and the combination of vitamins A, C, E, folate, and zinc. Therefore, the following protocol has been recommended by the board of physicians.

Controlled ovarian hyperstimulation was performed with antagonist protocol as following: the cycle was initiated with 150 IU/day recombinant follicle‐stimulating hormone (Gonal F; Serono) on Day 2 of the menstrual cycle. When at least one follicle reached ≥14 mm in diameter, 0.25 mg GnRH antagonist (Cetrotide; Merck Serono) was started and continued until the day of human chorionic gonadotropin (hCG) administration. A single dose of 10,000 IU hCG (Pregnyl; Organon) was administered when at least two follicles reached a mean diameter of 18 mm, then followed by oocyte retrieval 34 h later. Seven of eight retrieved oocytes were metaphase II (MII, matured). Despite the oligozoospermia condition in the former semen analysis, no sperm was found in the semen of the partner for ICSI. Therefore, based on the husband's decision not to perform TESE, all seven MII oocytes were vitrified to enable the couple to make future fertility decisions.

The vitrification of oocytes were performed according to the kitazato protocol (Kitazato Co.). Briefly, the oocytes were transferred into the basic solution (BS), then, 280 µl equilibration solution (ES) were slowly added to BS in three steps, in order to gradually facilitate the oocytes adjustment to the final concentration of cryoprotectants. After the completion of equilibration (14 min), the oocytes were transferred to vitrification solution (VS) for 1 min. Three and four oocytes were separately loaded on 2 cryotops (Kitazato Co.), afterward stored in liquid nitrogen (LN2).

Since sperm donation is not an acceptable alternative for infertility treatment in our country due to legislation and religion, the husband was subjected to TESE, which revealed immotile sperm with severe morphological abnormalities. The couple was informed of the minimal chances of fertilization and pregnancy, and the TESE sample was frozen with the patient's permission.

For freezing, dissected testicular sample was mixed with CryoSpermTM (Origio) with a 1:1 (v/v) ratio. After 10 min remaining at room temperature, the mixture was loaded in cryovials. The cryovials were placed in the vapor phase above the LN2 for 30 min and stored transferred into LN2.

Two months later, the couple decided to pursue their infertility treatment. Endometrium preparation was initiated from the second day of the menstrual cycle with the consumption of oral estradiol valerate (Aburaihan Co) 6 mg per day until endometrial thickness reached ≥8 mm and a triple line pattern in ultrasonography. A daily injection of 100 mg progesterone in oil (Aburaihan, Co) was started and continued to the 10th week of gestation. On the first day of progesterone administration, all the vitrified oocytes were warmed with a 100% survival rate.

Thawing of vitrified oocytes was performed based on Kitazato Co protocol: The oocytes were placed in warming solution 1 min at 37°C; then in dilution and washing solutions for 3 and 5 min, respectively. Following a wash in fresh medium, the oocytes were incubated for 2 h in GIVF (Vitrilife) media at 37°C, 6% CO_2_, and 5% O_2_.

**FIGURE 1:**
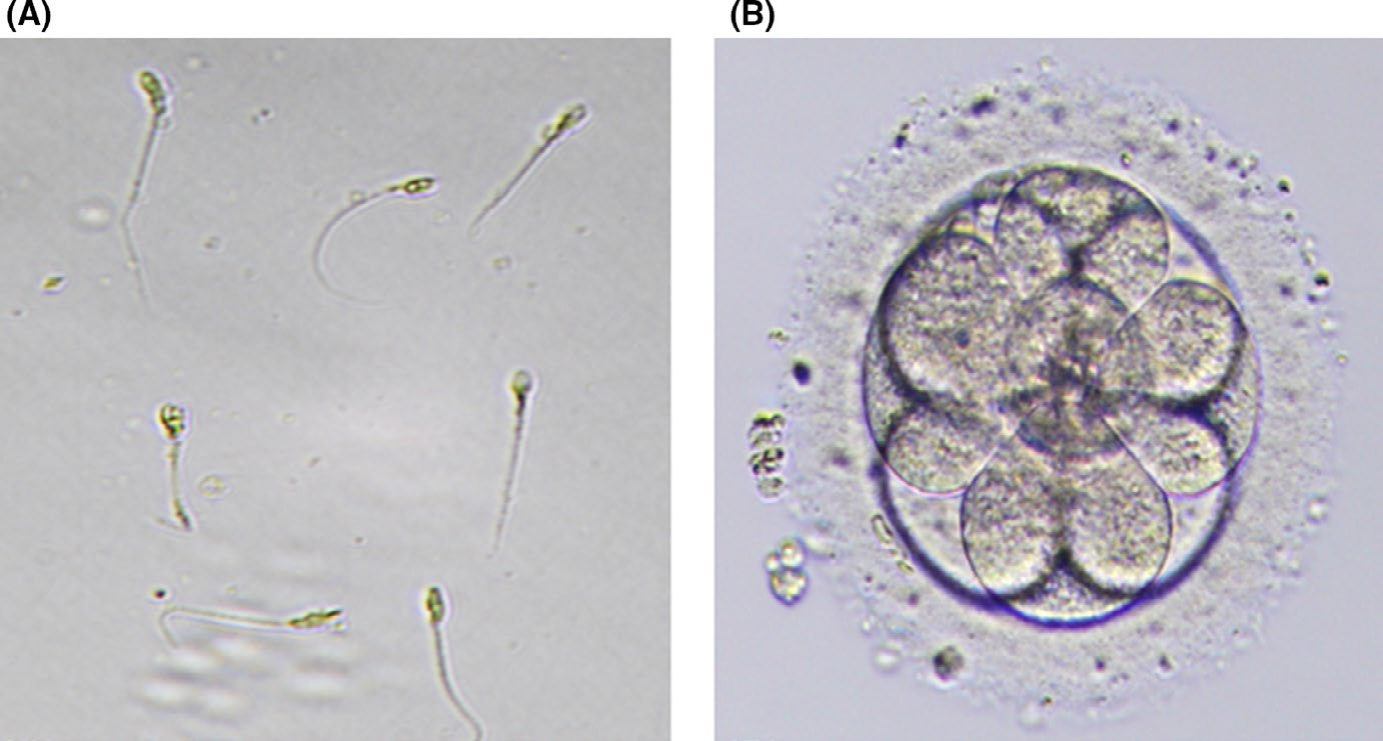
(A) Selected and injected sperms; (B) Day 3 embryo before transfer

Testicular sperm extraction sample was also thawed and prepared for ICSI. Only seven immotile sperms with severe morphological abnormalities and large vacuoles were obtained despite a four‐hour intensive search (Figure 1A). Desperately, the seven warmed oocytes were injected with these low‐quality sperms, and 17 h later assessed for the presence of pronuclei that one of them was fertilized and formed a good quality embryo, nine cells with grade A (Figure 1B), on Day 3 that resulted in pregnancy. A healthy female infant weighing 3380 gr was born at 39 weeks of gestation by caesarian section.

## 3 DISCUSSION

Currently, receiving a third person's sperm for infertility treatment has been approved in some circumstances, such as azoospermic men and single or homosexual women, but no decision has been made on this issue in other countries with a religious law such as Iran.^4^

Testicular sperm cryopreservation before ICSI is routinely performed in patients with obstructive azoospermia (OA) and NOA.^5^ Since in preliminary evaluation of this couple, the male partner was not diagnosed with azoospermia, and in previous failed ICSI/ET cycle, ejaculated sperms were used in the fresh oocyte cycle, based on the patient's informed refusal of recommended TESE, testicular sperm cryopreservation prior ICSI was not performed.

Intracytoplasmic sperm injection with frozen‐thawed testicular sperms, even in the fresh oocytes cycle, resulted in a low fertilization rate.^6^, ^7^ Live births are achieved by either using vitrified‐warmed oocytes with frozen‐thawed testicular sperms^6^, ^8^ or frozen‐thawed oocytes by slow freezing and frozen‐thawed testicular sperms; however, there appears to be limited reports in the literature. In all of these reports, injected sperms were motile with an acceptable number for ICSI. For example, Setti et al., reported a case that the frozen‐thawed testicular sperms of an obstructive azoospermic patient with relatively intact spermatogenesis were used for ICSI.^7^, ^9^ Selman's findings also show that when vitrified oocytes are combined with frozen testicular spermatozoa in eleven patients, viable embryos leading to healthy babies were produced.^8^

The success rate of TESE/ICSI cycles is highly dependent on the type of azoospermia, obstructive or non‐obstructive, and the availability of motile sperms.^6^ Lower fertilization and pregnancy rates were achieved after ICSI with testicular sperms from men with NOA compared with obstructive azoospermic males.^10^, ^11^ Presences of motile sperms in the fresh or frozen‐thawed ejaculate or TESE are necessary for optimal fertilization and pregnancy outcomes.^12^ Frozen testicular samples further reduce the availability of motile sperms.^6^

In one case reported by Zhang et al. in 2017, the patient did not achieve pregnancy following injection of fresh oocytes with very low counts, immotile ejaculated sperms. However, after injection of the vitrified‐warmed sibling oocytes, with frozen‐thawed donor sperms, the pregnancy and live birth were achieved.^1^ Similar to this case, when the ejaculated sperms or TESE sample have poor quality or resulted in unsatisfactory clinical outcomes, the use of a third person's sperm is recommended in almost all IVF centers worldwide.^13^

Assisted reproductive technology therapies are offered to infertile couples in ways shaped by their local considerations, including their social, cultural, religious, economic, ethical, and political circumstances. In this case, we had to inject immotile sperms with a severe morphological abnormality due to the limitation of the use of the donor sperm. Therefore, this case report demonstrates that viable offspring can be obtained from ICSI of vitrified‐warmed oocytes even with severely poor quality frozen‐thawed testicular sperms in couples who desire to have their genetic offspring.

## CONFLICT OF INTEREST

The authors declare no conflict of interest.

## AUTHOR CONTRIBUTIONS

Somayyeh Safari, who was the embryologist involved in the laboratory phase of the IVF cycle treatment, revised the manuscript and approved the final submission. Hoora Amouzegar, was the physician involved in patient care, revised the manuscript and approved the final submission. Sareh Ashourzadeh was involved in drafting the initial manuscript, revising the manuscript, and approved the final submission. Elham Hosseini involved in drafting the initial manuscript, editing the manuscript, and approved the final submission. All authors contributed substantially to the interpretation of the procedures in this case.

## ETHICAL APPROVAL

None.

## CONSENT

Written informed consent was obtained from the patient to publish this report in accordance with the journal's patient consent policy.

## Data Availability

All data underlying the results are available as part of the article, and no additional source data are required.
